# Are we sure that the neurological impact of COVID 19 in childhood has not been underestimated?

**DOI:** 10.1186/s13052-021-01144-y

**Published:** 2021-09-18

**Authors:** Nicola Principi, Susanna Esposito

**Affiliations:** 1 Università degli Studi di Milano, Milan, Italy; 2 Pediatric Clinic, Department of Medicine and Surgery, Pietro Barilla Children’s Hospital, University of Parma, via Gramsci 14, 43126 Parma, Italy

**Keywords:** ADEM, Children, COVID-19, MIS-C, Neurologic complications

## Abstract

**Background:**

Presently, it is known that, even if less frequently than in adults, children can develop a severe new coronavirus disease 2019 (COVID-19). Children with the SARS-CoV-2 infection can have neurological signs and symptoms of disease more frequently than previously thought, revealing the involvement of the central nervous system, the peripheral nervous system, or both. Aim of this manuscript is to highlight the neurologic complications associated with SARS-CoV-2 among pediatric patients with COVID-19, suggesting when to monitor carefully neurologic development.

**Main findings:**

Children with a severe chronic underlying disease, infants and toddlers and those who develop the so-called multisystem inflammatory syndrome (MIS-C) are those with the highest incidence of neurological complications. Fortunately, in most of the cases, neurological manifestations, mainly represented by headache and anosmia, are mild and transient and do not significantly complicate the COVID-19 course. However, in some cases, very severe clinical problems associated with relevant alterations of neuroimaging, electroencephalography, nerve conduction studies and electromyography findings can develop. Generally, almost all the children with COVID-19 and neurological manifestations till now described have made a complete recovery, although in some cases this has occurred after several weeks of treatment. Moreover, COVID-19 infection during pregnancy has been found associated with an increased risk of obstetric complications that can lead to neurological acute and long-term manifestations in neonates.

**Conclusions:**

Based on data showing the neurologic impact of COVID-19 in pediatric age, we suggest monitoring neurological development a few months after healing in pediatric patients who have presented MIS-C, seizures or other neurological manifestations and in children of pregnant women with COVID-19 in order to detect overt and subtle deficits.

## Background

In the first months of new coronavirus disease 2019 (COVID-19) pandemic, results of several epidemiological studies had led to the conclusion that, compared to adults, children were less susceptible to severe acute respiratory syndrome coronavirus-2 (SARS-CoV-2) infection and that, when infected, they remained more frequently asymptomatic or developed a mild disease [1–3]. Most of the symptomatic children had only minor respiratory or gastrointestinal problems. Moreover, the risk of hospitalization and the need for intensive care unit (ICU) admission was found very low and death was an exceptional event [4, 5]. These findings explain why pediatric COVID-19 was initially considered a minor clinical problem.

However, with the prolongation of the pandemic and a more accurate evaluation of a greater number of children, this belief was, at least in part, changed. Presently, it is known that, even if less frequently than in adults, children can develop a severe COVID-19. At higher risk are subjects with severe underlying medical conditions, younger children and those that develop the so-called multisystem inflammatory syndrome (MIS-C) [1, 6]. Moreover, the review of the clinical picture of pediatric COVID-19 has shown that its impact extends beyond the pulmonary morbidity and mortality of the disease. More frequently than previously thought, children with the SARS-CoV-2 infection can have neurological signs and symptoms of disease, revealing the involvement of the central nervous system (CNS), the peripheral nervous system (PNS), or both [1]. Their presence has prognostic significance and their impact could be fully clarified after many years of careful follow-up, and not only in the acute and subacute phase of the infection [1].

Aim of this manuscript is to highlight the neurologic complications associated with SARS-CoV-2 among pediatric patients with COVID-19, suggesting when to monitor carefully neurologic development.

## Neurologic complications associated to COVID-19 in pediatric age

Children with a severe chronic underlying disease, infants and toddlers and those who develop the so-called MIS-C are those with the highest incidence of neurological complications [7–9]. A systematic review and meta-analyses of studies involving about 3700 pediatric COVID-19 patients have shown that 17% of them had nonspecific neurologic conditions and 1% had signs and symptoms of severe brain involvement [7]. In MIS-C patients, the incidence of abnormal neurological findings has been reported in up to 55% of the cases [8]. Fortunately, in most of the children, neurological manifestations, mainly represented by headache and anosmia, are mild and transient and do not significantly complicate the COVID-19 course. However, in some cases, very severe clinical problems associated with relevant alterations of neuroimaging, electroencephalography, nerve conduction studies and electromyography findings can develop. A not marginal number of encephalopathy, encephalitis, central or peripheral acute flaccid paralysis, acute disseminated encephalomyelitis, seizures, stroke has been diagnosed in children with laboratory confirmed SARS-CoV-2 infection [9].

These findings seem to confirm what have been suggested by the studies involving other human coronaviruses (hCoVs), i.e. the risk that pediatric infections due to this group of agents may be associated with the development of neurological insults and that these may lead to a significant neurodevelopmental impairment [10]. Except for Middle East Respiratory Syndrome (MERS)-CoV, for all the other known hCoVs neurological complications have been described. In case of SARS-CoV-2 infection, the risk seems further increased by the evidence that neurological manifestations can develop both during the acute phase of COVID-19 as the first signs and symptoms of disease in previously healthy children and over days to weeks after a previously asymptomatic SARS-CoV-2 infection. In both cases, diagnosis and potentially effective therapy may be severely delayed or completely missed with high risk of persistent neurologic damage.

## Mechanisms that cause COVID-19-related neurologic damage

Several mechanisms have been postulated to explain acute and postacute neurologic manifestations of COVID-19. Although neurologic damage can simply be the consequence of hypoxia, hypotension and liver and renal insufficiency that are characteristic of the most severe cases of COVID-19, a number of specific mechanisms are considered the main causes of CNS and PNS damage [11]. It has been supposed that SARS-CoV-2 may infect olfactory neurons and subsequently invade brainstem, basal ganglia and cortex, with a direct destructive effect [11]. Moreover, as SARS-CoV-2 depletes Angiotensin-Converting Enzyme 2 (ACE-2) causing a significant renin-angiotensin system disequilibrium, it is thought that, following the infection, a prothrombotic state with impaired large vessel and microvascular blood flow can occur and lead to increased risk of thrombotic and hemorrhagic stroke [11]. The most important mechanism seems, however, the immune dysregulation leading to autoimmunity and the hyperinflammation [11]. Most cases occur in children with MIS-C and most neuroimaging findings resemble those detected in parainfectious and postinfectious syndromes that involve the CNS and the PNS [12].

Moreover, in some patients with acute disseminated encephalomyelitis (ADEM)-like magnetic resonance images, antibodies against brain components have been detected, strongly suggesting that COVID-19 can be associated with an immune-mediated pathology [13]. However, regardless of the pathophysiology, in all these cases, early identification, prompt diagnosis and careful implementation of the most effective therapeutic interventions are needed to assure the resolution of the acute disease and the potential long-term abnormalities.

## Outcome of COVID-19-related neurologic complications in pediatric age

Generally, almost all the children with COVID-19 and neurological manifestations till now described have made a complete recovery, although in some cases this has occurred after several weeks of treatment [7–9]. However, definitive conclusions about long-term consequences cannot be drawn due to the low number of study cases and the time-limited follow-up. On the other hand, the risk that, at least in some cases, some long-term neurological problems may persist cannot be excluded. When prognosis of the clinical conditions described in COVID-19 pediatric cases is analyzed, it can be observed that some of them can cause long-term problems [13]. An example in this regard is given by ADEM. Generally, ADEM cases have complete resolution. However, a recent meta-analysis has found that, despite a generally positive neurocognitive outcome, a reduction of intelligence quotient, attention, executive functioning, processing speed, learning and memory, visuospatial skills and internalizing symptoms could persist in up to 43% subset of individuals several months after the apparent resolution of the disease [12]. This explains why experts have suggested that COVID-19 is included among the medical conditions that can potentially cause long-term neurological problems and that children with SARS-CoV-2 infection even without neurological manifestations in the acute phase of disease should be carefully monitored for a long time. To favor the early identification of potential neurodevelopmental impairment, the use of an age-related clinical practice guidance for pediatric providers treating children with SARS-CoV-2 infections has been proposed [9].

The importance of monitoring children involved in the problem of SARS-CoV-2 in-fection is further highlighted by the recently acquired evidence that infection of the pregnant women can have a relevant negative impact on the neonates [14, 15]. Maternal infections during the second and the third trimester of pregnancy have been frequently found associated with an increased risk of obstetric complications leading to preterm birth, low birth weight and several neonatal diseases, including respiratory distress syndrome, hyperbilirubinemia and need for assistance in ICU [14]. As all these conditions can lead to neurological acute and long-term manifestations, implications of SARS-CoV-2 as a cause of neurological problems in children are significantly more important than those simply due to the direct infection of the child.

## Conclusions

Based on data on neurological involvement in SARS-CoV-2 positive pediatric patients and on neonatal complications observed in children of pregnant women with COVID-19, we suggest monitoring neurological development a few months after healing in pediatric patients who have presented MIS-C, seizures or other neurological manifestations and in children of pregnant women with COVID-19 in order to detect overt and subtle deficits. As the focus of healthcare shifts from the ‘crisis’, diligence, attention and multicentric collaborations will be required to ensure ongoing international collaboration in maintaining registries to monitor for possible later behavioral complications of COVID-19 in children who have being infected, even without clinical evidence of neurological signs and symptoms. In particular, it will be important to evaluate subtle neurobehavioral impairment which can be evident only many years later because of the known SARS-CoV- 2 localization in certain cerebral areas linked to neurobehavioral development skills (i.e., orbito-frontal and pre-frontal areas).

In addition, during the pandemic, the chronic-relapsing neurological pathology (i.e., epilepsy) was neglected by many families due to the fear of being infected by SARS-CoV-2 with access to the Emergency Room and being subjected to diagnostic delays related to COVID-19 before hospitalization (which often also meant strict isolation and estrangement from the family). Thus, it is crucial that management of these patients could return to previous clinical practice since this could avoid that they are admitted to the Emergency Room in extreme conditions.

## Data Availability

All included.

## Abbreviations

ACE-2: Angiotensin-Converting Enzyme 2
ADEM: acute disseminated encephalomyelitis
CNS: central nervous system
COVID-19: new coronavirus disease 2019
hCoVs: human coronaviruses
ICU: Intensive Care Unit
MERS: Middle East Respiratory Syndrome
MIS-C: multisystem inflammatory syndrome in children
PNS: peripheral nervous system
SARS-CoV-2: severe acute respiratory syndrome coronavirus

## References

[1] Dong Y, Mo X, Hu Y, Qi X, Jiang F, Jiang Z, Tong S (2020). Epidemiology of COVID-19 among children in China. Pediatrics..

[2] Vergine G, Fantini M, Marchetti F, Stella M, Valletta E, Biasucci G, Lanari M, Dodi I, Bigi M, Magista AM, Vaienti F, Cella A, Affanni P, Re MC, Sambri V (2020). Esposito S; Regione Emilia-Romagna COVID-19 Pediatric Working Group (RERCOPed). Home Management of Children With COVID-19 in the Emilia-Romagna Region, Italy. Front Pediatr.

[3] Esposito S, Marchetti F, Lanari M, Caramelli F, De Fanti A, Vergine G, Iughetti L, Fornaro M, Suppiej A, Zona S, Pession A, Biasucci G (2021). Working group on COVID-19 in pediatrics of the Emilia-Romagna region (RE-CO-Ped). COVID-19 Management in the Pediatric age: consensus document of the COVID-19 working Group in Paediatrics of the Emilia-Romagna region (RE-CO-Ped), Italy. Int J Environ Res Public Health.

[4] Götzinger F, Santiago-García B, Noguera-Julián A, Lanaspa M, Lancella L, Calò Carducci FI, Gabrovska N, Velizarova S, Prunk P, Osterman V, Krivec U, Lo Vecchio A, Shingadia D, Soriano-Arandes A, Melendo S, Lanari M, Pierantoni L, Wagner N, L'Huillier AG, Heininger U, Ritz N, Bandi S, Krajcar N, Roglić S, Santos M, Christiaens C, Creuven M, Buonsenso D, Welch SB, Bogyi M, Brinkmann F, Tebruegge M, Pfefferle J, Zacharasiewicz A, Berger A, Berger R, Strenger V, Kohlfürst DS, Zschocke A, Bernar B, Simma B, Haberlandt E, Thir C, Biebl A, vanden Driessche K, Boiy T, van Brusselen D, Bael A, Debulpaep S, Schelstraete P, Pavic I, Nygaard U, Glenthoej JP, Heilmann Jensen L, Lind I, Tistsenko M, Uustalu Ü, Buchtala L, Thee S, Kobbe R, Rau C, Schwerk N, Barker M, Tsolia M, Eleftheriou I, Gavin P, Kozdoba O, Zsigmond B, Valentini P, Ivaškeviciene I, Ivaškevicius R, Vilc V, Schölvinck E, Rojahn A, Smyrnaios A, Klingenberg C, Carvalho I, Ribeiro A, Starshinova A, Solovic I, Falcón L, Neth O, Minguell L, Bustillo M, Gutiérrez-Sánchez AM, Guarch Ibáñez B, Ripoll F, Soto B, Kötz K, Zimmermann P, Schmid H, Zucol F, Niederer A, Buettcher M, Cetin BS, Bilogortseva O, Chechenyeva V, Demirjian A, Shackley F, McFetridge L, Speirs L, Doherty C, Jones L, McMaster P, Murray C, Child F, Beuvink Y, Makwana N, Whittaker E, Williams A, Fidler K, Bernatoniene J, Song R, Oliver Z, Riordan A (2020). COVID-19 in children and adolescents in Europe: a multinational, multicentre cohort study. Lancet Child Adolesc Health.

[5] Esposito S, Caramelli F, Principi N (2021). What are the risk factors for admission to the pediatric intensive unit among pediatric patients with COVID-19?. Ital J Pediatr.

[6] McArdle AJ, Vito O, Patel H, Seaby EG, Shah P, Wilson C, Broderick C, Nijman R, Tremoulet AH, Munblit D, Ulloa-Gutierrez R, Carter MJ, De T, Hoggart C, Whittaker E, Herberg JA, Kaforou M, Cunnington AJ, Levin M, BATS Consortium (2021). Treatment of multisystem inflammatory syndrome in children. N Engl J Med.

[7] Panda PK, Sharawat IK, Panda P, Natarajan V, Bhakat R, Dawman L. Neurological Complications of SARS-CoV-2 Infection in Children: A Systematic Review and Me-ta-Analysis. J Trop Pediatr. 2020; Epub Sep 10:fmaa070. doi: 10.1093/tropej/fmaa070, 2021. 10.1093/tropej/fmaa070 PMC7499728 32910826

[8] Esposito S, Principi N (2021). Multisystem inflammatory syndrome in children related to SARS-CoV-2. Paediatr Drugs.

[9] Singer TG, Evankovich KD, Fisher K, Demmler-Harrison GJ, Risen SR (2021). Coronavirus infections in the nervous system of children: a scoping review making the case for long-term neurodevelopmental surveillance. Pediatr Neurol.

[10] Principi N, Bosis S, Esposito S (2010). Effects of coronavirus infections in children. Emerg Infect Dis.

[11] Schober ME, Pavia AT, Bohnsack JF. Neurologic manifestations of COVID-19 in children: emerging pathophysiologic insights. Pediatr Crit Care Med. 2021; Epub May 7. doi: 10.1097/PCC.0000000000002774. 10.1097/PCC.0000000000002774 PMC8240497 33965992

[12] Lindan CE, Mankad K, Ram D, Kociolek LK, Silvera VM, Boddaert N, Stivaros SM, Palasis S, Akhtar S, Alden D, Amonkar S, Aouad P, Aubart M, Bacalla JA, Barbosa AA, Basmaci R, Berteloot L, Blauwblomme T, Brun G, Carney O, Chareyre J, Chéron G, Coimbra PPDA, Dangouloff-Ros V, D'Arco F, Dineen R, de-Pontual L, Desguerre I, Elfallal W, Evans DG, Ferraciolli SF, Girard N, Gonçalves FG, Gonzalez I, Grant PE, Grévent D, Guimaraes CVA, Hassell J, Hirata FCC, Kamaly-Asl I, Jacob J, Jackson K, Jones BV, Joseph R, Jung AY, Kashgari A, Kilday JP, Kirsch A, Kossorotoff M, Krishnan A, Kulkarni S, Leruez-Vill M, Lesage F, Levy R, Li Y, Lima CCV, Lingappa L, Löbel U, Lopez-Alberola R, Lucato LT, Moreira DD, Murnick JG, Nahmani S, Pagariya S, Pavaine J, Philbrook B, Piovesan AC, Poisson KE, Reddy N, Riley P, Romsauerova A, Roux CJ, Rugilo C, Saigal G, Salvador GLO, Seidenwurm D, Sermet-Gaudelus I, Sidpra J, Sudhakar SV, Toronchik MS, Vézina G (2021). Neu-roimaging manifestations in children with SARS-CoV-2 infection: a multinational, Mul-ticentre collaborative study. Lancet Child Adolesc Health..

[13] Siracusa L, Cascio A, Giordano S, Medaglia AA, Restivo GA, Pirrone I, Saia GF, Collura F, Colomba C (2021). Neurological complications in pediatric patients with SARS-CoV-2 infection: a systematic review of the literature. Ital J Pediatr.

[14] Dumitriu D, Gyamfi-Bannerman C (2021). Understanding risk for newborns born to SARS-CoV-2–positive mothers. JAMA..

[15] Lassi ZS, Ana A, Das JK, Salam RA, Padhani ZA, Irfan O, Bhutta ZA (2021). A systematic review and meta-analysis of data on pregnant women with confirmed COVID-19: clinical presentation, and pregnancy and perinatal outcomes based on COVID-19 severity. J Glob Health.

